# The complete chloroplast genome sequence of *Taxillus yadoriki* (Loranthaceae): a hemi-parasitic evergreen shrub in East Asia

**DOI:** 10.1080/23802359.2020.1806755

**Published:** 2020-08-17

**Authors:** Won-Bum Cho, Eun-Kyeong Han, Dong Chan Son, Jung-Hyun Lee

**Affiliations:** a Department of Biology Education, Chonnam National University, Gwangju, Republic of Korea; b Division of Forest Biodiversity and Herbarium, Korea National Arboretum, Pocheon, Republic of Korea

**Keywords:** Chloroplast genome, gene loss, phylogenetic analysis, *Taxillis*

## Abstract

*Taxillus yadoriki* (Loranthaceae) is a hemiparasitic evergreen shrub distributed in Korea and Japan. We report the complete chloroplast genome of *T. yadoriki* to provide insight into the phylogenetic relationship of Loranthaceae. This genome is 122,192 bp long, with two IR regions (22,756 bp each) that separate a large single-copy (LSC) region (70,628 bp) and a small single-copy (SSC) region (6052 bp). It contains 109 genes that encode 68 proteins, 8 rRNAs, and 33 tRNAs. All of *ndh* genes have been lost and the SSC region consists of only four genes similar to other *Taxillus* species. In ML phylogenetic, monophyly of *Taxillus* was strongly supported with high bootstrap value and formed a sister group with *Scurrula.*

*Taxillus* L., comprises approximately 30 species that are widely distributed, with the majority occurring on the warm-temperate and subtropical regions in Asia (Wu et al. 2003). The genus displays a lifestyle as hemiparasites, specialized by parasitic organs called haustoria (Li et al. 2017). The chloroplast genome (cp) of *Taxillus* has undergone structural changes and gene loss that play a key role in the evolution from autotrophy to parasitism (Li et al. 2017; Zhao et al. 2019). However, to date, only a few plastid genome (plastome) has been sequenced for *Taxillus*.

*Taxillus yadoriki* (Maxim.) Danser (Loranthaceae) is a hemiparasitic evergreen shrub distributed in Korea and Japan (Ohba 2006). Also, the species is a very important medical plant with various pharmacological activities such as anti-cancer, anti-inflammation (Park et al. 2018). Thus, we characterized the chloroplast genome of *T. yadoriki* and analyzed its phylogenetic position within Loranthaceae.

In this study, material of *T. yadoriki* were collected from the Jeju Island in South Korea (N 33°15′23", E 126°37′23"). The voucher specimen (Lee. 2005039) was stored at the herbarium in the Department of Biology Education, Chonnam National University (BEC). The DNA library was constructed and sequenced using MGI-seq 2000 platform (LAS, Seoul, Korea), generating 34,340,232 raw reads (150 bp paired-end). After trimming the sequences, the obtained clean data were mapped with the reference cp genome for *T. sutchunensis* (NC.036037), using Geneious 10.2.3 (Kearse et al. 2012). On that genome, 3,926,088 reads were assembled with an average of 4808X coverage (max: 6916X, min: 1146X). The annotation was separately performed using DOGMA (Wyman et al. 2004), and were manually corrected for start and stop codons and for intro/exon boundaries. The annotated cp genome sequence was deposited in the GenBank with Accession Number MT702883. To construct the phylogenetic tree, we downloaded complete cp genome sequences of 9 related species from the NCBI database and aligned using the MAFFT (Katoh and Toh 2010). We performed maximum likelihood (ML) analysis with RAxML v.8.0 (Stamatakis 2014) using default parameters and 1000 bootstrap replicates.

The cp genome structure of *T. yadoriki* is 122,192 bp long, with 2 IR regions (22,756 bp each) that separate a large single-copy (LSC) region (70,628 bp) and a small single-copy (SSC) region (6,052 bp). Overall G + C content of the genome is 37.3%, compared with 34.6% for the LSC and 42.8% for the IRs. This cp genome contains 109 genes that encode 68 proteins, 8 rRNAs, and 33 tRNAs. All of the *ndh* genes have been lost and the SSC region consists of only four genes (*trn*L^UAG^, *ccs*A, *psa*C, *ycf*1) similar to other *Taxillus* species (Li et al. 2017). The *trn*L^UAG^ was located on the IR_A_ and SSC region border similar to other Loranthaceae species. In ML phylogenetic, monophyly of *Taxillus* was strongly supported with high bootstrap value (100%) and formed a sister group with *Scurrula* (Figure 1). *Taxillus yadoriki* is sister to a clade consisting of *T. nigrans*, *T. vestitus* and *T. sutchuenensis*. These new phylogenetic data provide insight into the evolutionary progress of Loranthaceae. 

**Figure 1.**
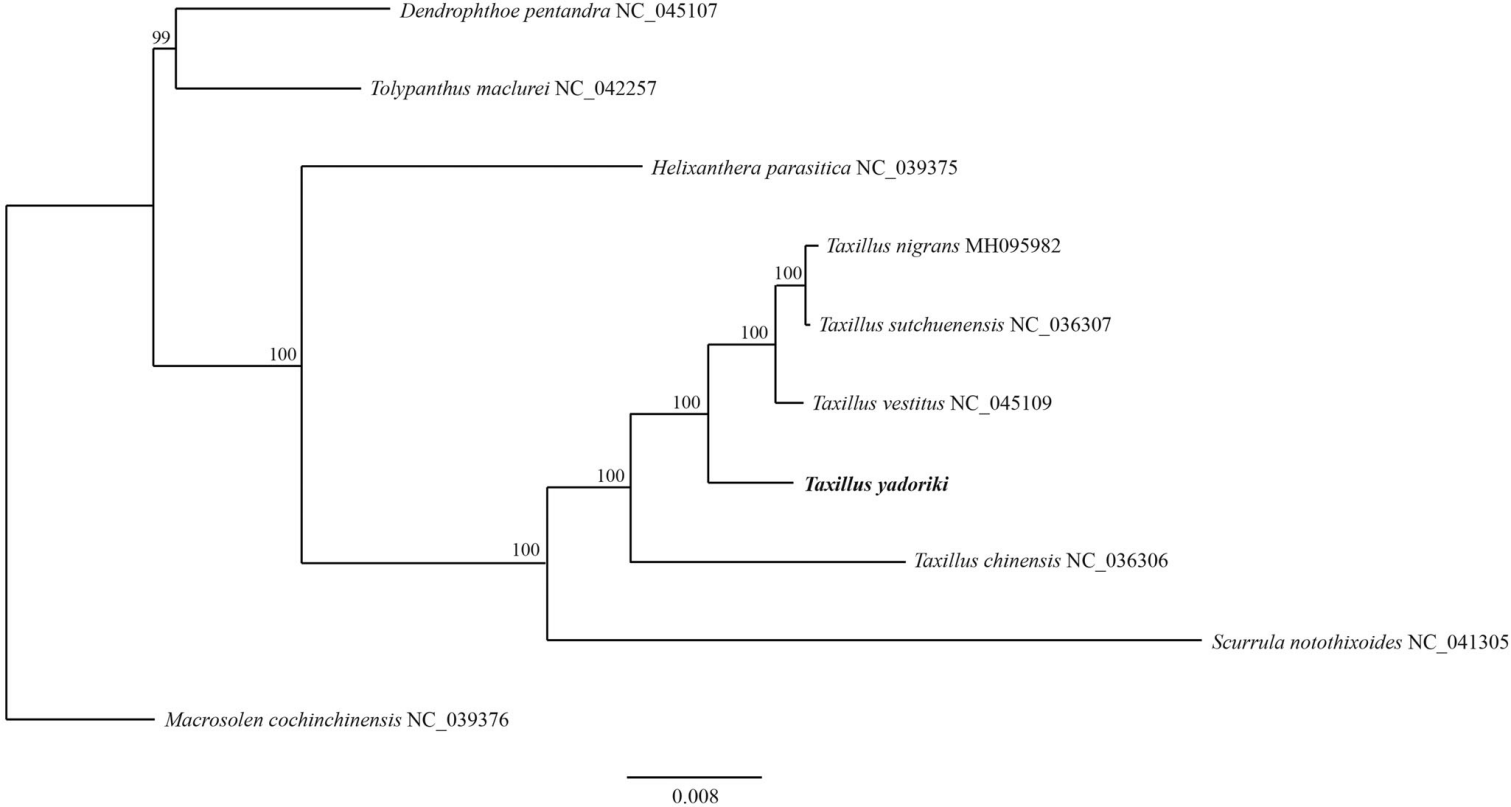
Maximum-likelihood phylogenetic tree was constructed based on 10 complete cp genome sequences of Loranthaceae. Numbers above nodes indicate bootstrap values with 1,000 replicates.

## Data Availability

The data that support the findings of this study are openly available in GenBank of NCBI (accession no. MT702883) at https://www.ncbi.nlm.nih.gov.
